# Influence of Diet and Nutrition on Prostate Cancer

**DOI:** 10.3390/ijms21041447

**Published:** 2020-02-20

**Authors:** Makoto Matsushita, Kazutoshi Fujita, Norio Nonomura

**Affiliations:** Department of Urology, Osaka University Graduate School of Medicine, Suita, Osaka 565-0871, Japan; matsushita@uro.med.osaka-u.ac.jp (M.M.); nono@uro.med.osaka-u.ac.jp (N.N.)

**Keywords:** prostate cancer, nutrition, diet, gut microbiota

## Abstract

The incidence of prostate cancer (PCa) displays widespread regional differences, probably owing to differences in dietary habits. Nutrients, including fat, protein, carbohydrates, vitamins (vitamin A, D, and E), and polyphenols, potentially affect PCa pathogenesis and progression, as previously reported using animal models; however, clinical studies have reported controversial results for almost all nutrients. The effects of these nutrients may be manifested through various mechanisms including inflammation, antioxidant effects, and the action of sex hormones. Dietary patterns including the Western and Prudent patterns also influence the risk of PCa. Recent studies reported that the gut microbiota contribute to tumorigenesis in some organs. Diet composition and lifestyle have a direct and profound effect on the gut bacteria. Human studies reported an increase in the abundance of specific gut bacteria in PCa patients. Although there are few studies concerning their relationship, diet and nutrition could influence PCa, and this could be mediated by gut microbiota. An intervention of dietary patterns could contribute to the prevention of PCa. An intervention targeting dietary patterns may thus help prevent PCa.

## 1. Introduction

Prostate cancer (PCa) is the second most common type of cancer and the fifth most common cause of cancer-related death in men worldwide [1]. In 2018, ~1.2 million new cases of PCa were diagnosed, and nearly 360,000 men died of PCa worldwide [1]. The incidence of PCa typically varies widely in residential areas and is ~6-fold higher in Western countries than in non-Western countries [2].

One reason for the variability in PCa incidence in residential areas concerns innate factors, such as racial differences. African-Americans have the highest rate of PCa, followed by Caucasians, whereas Asians have the lowest. In general, there is a threefold difference between Asians and African-Americans [3]. These results are likely related to differences in genetic variation and polymorphism among races; however, Japanese men who have emigrated to Western countries have an increased incidence of PCa relative to Japanese residents in Japan [4,5]. This suggests that both innate factors and environmental factors, such as lifestyle, can influence PCa development.

Various environmental factors, such as diet, obesity, smoking, and exercise, are reportedly associated with PCa. Tobacco smoking is a risk factor in many types of cancer, and similarly, in PCa, smoking is associated with a significantly increased risk of overall mortality, cancer-specific mortality, and recurrence [6,7]. Exercise therapy often results in physical and mental improvements. According to an analysis of 752 Canadians, an active lifestyle reduced PCa risk [odds ratio (OR) = 0.8, 95% confidence interval (CI) = 0.6–0.9] [8]; however, there was no clear association between exercise and PCa in a recent meta-analysis of 28,707 cases [relative risk (RR) = 1.00, 95% CI = 0.99–1.01] [9].

Obesity is reportedly associated with various cancers, including PCa [10,11] and is an independent predictor of PCa according to a prospective study of 3673 men in the United States [RR = 1.7 for body mass index (BMI) > 27.8 kg/m^2^ vs. BMI < 23.6 kg/m^2^] [12]. Additionally, several epidemiological studies strongly suggest obesity is associated with progression in advanced PCa [13]. The prevalence of obesity varies significantly across regions, ranging from almost 40% in the United States to <~10% in Asian countries [14]. Therefore, regional differences in obesity rates might contribute to differences in the incidence of high-risk PCa. Moreover, obesity resulting from a high-calorie diet causes chronic systemic inflammation and might be involved in PCa growth through immune system-related mechanisms [13,15].

Because PCa has long been thought to be associated with food, associations with various nutrients, such as fat, and dietary pattern, such as Western diets, have been reported. Recently, some reports indicated a relationship between PCa and gut microbiota, which is altered according to diet.

**Figure 1:**
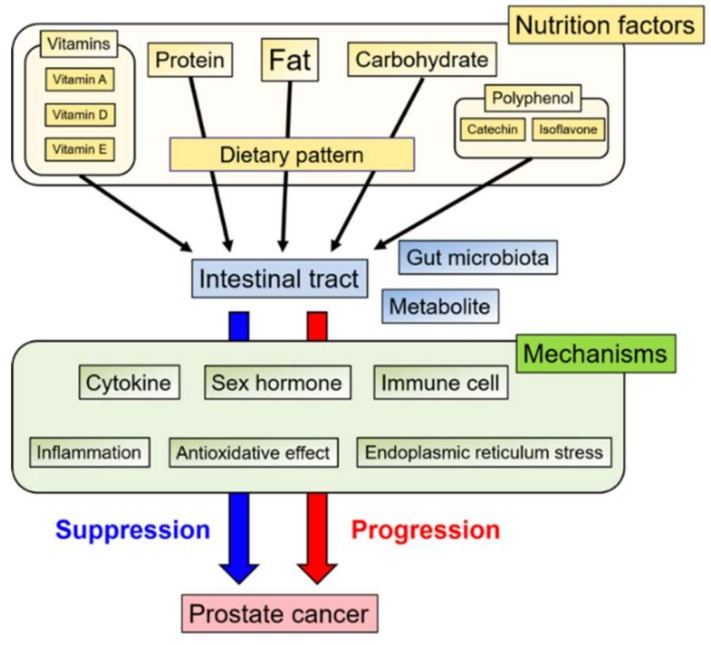
Schema of various nutrition factors and mechanisms involved in the progression or suppression of prostate cancer via the intestinal tract.

In this review, we summarize and discuss associations between PCa and diet. Additionally, we briefly describe the relationship between PCa and gut microbiota, which has received considerable attention in recent years and is being extensively studied (Figure 1).

## 2. Nutrition factors

### 2.1. Fat

Fatty acids are stored in the cytoplasm of many cells, mainly in the form of triacylglycerols, which comprise three fatty acid chains linked to a glycerol molecule. Triacylglycerols are found in food as animal fats, including meat and butter, and as plant oils, such as corn oil and olive oil. Fatty acids comprise a hydrophobic hydrocarbon chain and hydrophilic carboxylic acid and are classified as “saturated” (e.g., palmitic acid) or “unsaturated” (e.g., α-linolenic acid) according to the structure of the hydrophobic hydrocarbon chain. Fatty acids present in food are absorbed in the intestine and stored in cells as components of cell walls or energy sources, as they produce 6-fold more usable energy per gram than glucose when broken down [16].

Numerous cohort and case-control studies report that increased dietary fat intake is associated with PCa risk [17,18,19,20], although other studies showed no association [21,22,23,24]. Additionally, the relationship between total fat consumption and PCa is controversial. A population-based case-control study of 1,300 men aged <60 years in the United Kingdom reported that the risk of PCa increased among those with higher lipid intake as compared with those with lower lipid intake (OR = 2.53, 95% CI = 1.72–3.74) [20]. By contrast, basic research studies repeatedly show that fat consumption induces PCa growth, with several mechanisms identified as promoters of PCa development and progression in subjects fed a high-fat diet (HFD).

Obesity results in hyperinsulinemia and increased amounts of circulating bioactive insulin-like growth factor-1 (IGF-1), a growth factor that promotes the development of many types of cancer [25]. In PCa, proliferation is inhibited, and apoptosis is induced in LNCaP cells (a human prostate cancer cell line) cultured with sera from men fed a low-fat diet, with these findings accompanied by low levels of IGF-1 [26]. Additionally, mice fed a low-fat diet show significantly lower levels of serum prostate-specific antigen (PSA), serum insulin, and tumor *Igf1* mRNA levels relative to HFD mice, as well as a slower tumor-growth rate in LAPC4 xenografts [26,27]. Regarding the IGF-1 pathway, several studies demonstrate that IGF-I/phosphoinositide 3-kinase/AKT signaling plays an important role in fat-induced PCa development and progression [28].

Previous studies have focused on the relationship between fat-induced inflammation and PCa [29,30,31,32,33]. In a study using prostate-specific *phosphatase and tensin homolog* (*Pten*)^−/−^ mice, we found that an HFD doubled the tumor weight in the model mice but did not increase tumor weight in mice administrated celecoxib, a cyclooxygenase-2 (COX-2) inhibitor, or an anti-interleukin (IL)-6 antibody [29]. Further investigation showed that the mechanism involved local inflammation in the prostate gland induced by the HFD, which led to increased counts of tumor-infiltrating myeloid-derived suppressor cells and production of IL-6 by macrophages, thereby inducing PCa growth by suppression of tumor immunity and via the IL-6/signal transducer and activator of the transcription 3 (STAT3) signaling pathway [29]. A previous study showed that the STAT3 pathway plays an important role in HFD-induced PCa progression in an animal model [30], whereas another study investigating prostate tissue from obese PCa-model mice revealed elevated levels of C-X-C motif chemokine ligand (CXCL)12, C-X-C motif chemokine receptor (CXCR)4, and CXCR7 and showed that a CXCR4 agonist inhibited the migration and invasion of PCa cell lines treated with CXCL12 [31]. Additionally, in vivo and in vitro studies suggest that elevated monocyte chemoattractant protein 1 [also known as chemokine ligand (CCL2)] levels produced by adipocytes and elevated levels of the CCL2 receptor chemokine receptor 2 in prostate tissue are involved in HFD-induced PCa progression [32,33]. These results suggest a mechanism related to fat-induced PCa progression likely involves prostate-specific inflammation, suggesting roles for proinflammatory cytokines and chemokines.

HFD induces lipid accumulation in prostate tumors and promotes PCa metastasis. A previous study showed that aberrant sterol regulatory element-binding protein (SREBP)-dependent lipid metabolism is involved in PCa metastasis [34]. Epidemiological studies show that although high intake of saturated fatty acids (abundant in meat and butter) decrease the survival rate of PCa patients, unsaturated fatty acids (abundant in fish and vegetable oils) reportedly reduce the risk of PCa [35,36]. An animal study showed that mimicking MYC overexpression induces PCa progression in PCa-model mice fed a diet high in saturated fatty acids [37]. However, suppression of PCa cell proliferation by intake of unsaturated fatty acids, such as eicosapentaenoic acids, docosahexaenoic acids, and α-linolenic acids, was demonstrated in an animal study and several in vitro experiments using human PCa cell lines [38,39,40,41].

There is considerable epidemiological evidence that HFD affects hormone metabolism and might increase the risk of certain hormone-dependent diseases in men [42]. Sex hormones are important to PCa development and progression. Although reduction of serum cholesterol does not change PCa incidence in prostate-specific *Pten*^−/−^ mice, it lowers intraprostatic androgens and suppresses tumor progression [43]. Another study reported that consumption of high amounts of ω-3 fatty acid, an unsaturated fatty acid, slowed down prostate tumorigenesis by lowering estradiol, testosterone, and androgen receptor levels in transgenic mice [44]. These results suggest that a mechanism involved in fat-induced PCa progression might include regulation of sex hormones, in which unsaturated fatty acids play a specific role.

In summary, multiple studies indicate that excessive intake of saturated fatty acids and consequent obesity might contribute to PCa progression through multiple mechanisms, including inflammation and abnormal control of sex hormones and growth factors.

### 2.2. Protein

Because we cannot synthesize essential amino acids de novo, we must consume protein-containing food. In general, we consume proteins from at least one of three sources: animal meat, protein-rich plants, and dairy products.

Several clinical studies exploring the relationship between dietary protein intake and risk of PCa reported inconsistent findings. Recently, a meta-analysis of 12 articles comprising 13,483 PCa cases and 286,245 participants revealed that protein intake has no effect on PCa. In this report, the RR of the highest protein intake versus the lowest protein intake on PCa incidence was 0.993 (95% CI = 0.930–1.061). Further, there was no significant association between PCa risk and intake of animal or plant protein [45].

Regarding intake of animal meat and carcinogenesis, heterocyclic amines (HCAs) are formed when cooking all of the components in meat (e.g., creatine, amino acids, and sugar) at high temperatures and have been described as mutagenic compounds [46]. Previous studies analyzing the relationship between HCAs and men with PCa report well-cooked meat as being associated with increased PCa risk [47,48,49]. Exposure of rats to 2-amino-1-methyl-6-phenylimidazo [4,5-b] pyridine (PhIP), an HCA in well-cooked meat, demonstrated its role as a promoter of lobe-specific PCa [50,51]. Moreover, it was shown that mast cells and macrophages infiltrate the prostate gland in response to PhIP, suggesting that HCAs can increase PCa risk by promoting local inflammation [50].

Another protein source implicated in PCa carcinogenesis is dairy products, such as milk, butter, and cheese. Although epidemiological studies addressing dairy products and PCa have been conducted, their conclusions are controversial. A meta-analysis of 12 prospective studies reported an increased risk of PCa among men who consumed large amounts of dairy products (RR = 1.11, 95% CI = 1.00–1.22) and calcium (RR = 1.39, 95% CI = 1.09–1.77) [52], whereas another meta-analysis of 45 studies reported no association between consumption of dairy products and PCa (RR = 1.06, 95% CI = 0.92–1.22) [53]. Moreover, several clinical studies reported a positive association between high-fat milk intake and PCa progression and no association with low-fat milk intake [54,55], and a positive association between calcium overdose and the risk of PCa was reported [52,56,57]. Furthermore, an animal study using a mouse PCa model reported that high milk consumption exhibited slight protective effects against PCa progression by decreasing the expression of Ki-67 and G protein-coupled receptor family C group 6 member A [58]. Collectively, these results suggest that lipids and calcium might play a role in PCa carcinogenesis rather than proteins among the nutrients in dairy products. To verify these findings, the effects of high-fat milk and no-fat milk should be compared in PCa model mice.

### 2.3. Carbohydrates

Carbohydrates are abundant in plant-based foods, such as fruits, rice, wheat, and potatoes, and are mainly classified as monosaccharides, disaccharides, oligosaccharides, and polysaccharides according to the number of monomers in the molecule. Monosaccharides and disaccharides, which are abundant in fruits, dairy products, and sugar as glucose, fructose, and lactose, are rapidly metabolized in the body, and high intake can lead to hyperinsulinemia and obesity. As a result, high consumption of monosaccharides or disaccharides likely contributes to PCa progression through IGF-1 inflammation and activation [59].

Xenografted mice implanted with LAPC-4 cells fed a no-carbohydrate ketogenic diet (NCKD) showed a 15% reduction in body weight, a 33% reduction in tumor size, and an increase in overall survival as compared with mice fed a Western diet, with these results similar to or better than those for mice fed a low-fat diet [60]. Additionally, this study found that serum insulin, IGF-1, and the IGF-1:IGF-binding protein-3 ratio were lower among NCKD mice. Similar results were reported in a study using mice harboring xenografts involving LNCaP cells. Further, the phosphorylated-AKT:AKT ratio was reduced in tumors of NCKD mice relative to those fed an HFD/moderate-carbohydrate diet [28]. These results suggest that PCa progression induced by carbohydrate consumption is associated with obesity and hyperinsulinemia. Although many people find it difficult to sustain no-carbohydrate diets, which might be effective at reducing PCa risk, low-carbohydrate diets (20% carbohydrates, which might be sustainable for many people) show similar tumor-growth rates as those of no-carbohydrates diets [61].

There have been few human studies focused on the relationship between carbohydrate intake and PCa risk. In a prospective study of 3,184 adults from the Framingham Offspring cohort, the quantity and quality of carbohydrates and sugars were investigated in relation to obesity-related cancers (breast, prostate, and colorectal). They reported that higher intake of fruit juice was significantly associated with PCa risk (HR = 1.58, 95% CI = 1.04–2.41), whereas there was no significant association between PCa risk and total consumption of carbohydrate-heavy or sugary beverages [62,63]. However, a study comprising 22,720 men reported that sugar intake from fruit juice was unrelated to PCa risk, whereas intake of sugar-sweetened beverages was related to this risk (HR = 1.21, 95% CI = 1.06–1.39) [64]. These findings suggest that the quality of carbohydrates might be more important than their quantity regarding the relationship between carbohydrate consumption and PCa risk, although more research in future clinical trials is required. Analyses of the data from the National Health and Nutrition Examination Survey in the United States revealed that African-Americans drank more sugar-sweetened beverages [OR = 1.89] and fruit juices [OR = 2.73] than Caucasians [65], and the intake of sugar-sweetened beverages was the highest among adult African-American men (8.3% of total daily kilocalories), followed by Caucasians (6.4%), and lowest in Asians (4.0%) [66]. These results are consistent with the racial differences in PCa incidence, suggesting that the intake of harmful carbohydrates such as sugar-sweetened beverages may contribute to these differences.

### 2.4. Vitamins

Vitamins are small organic compounds other than carbohydrates, proteins, and lipids that cannot be synthesized in sufficient amounts de novo but are required for survival and growth. Therefore, humans need to obtain most of their vitamins from the diet or through supplements [16]. The number of vitamins varies from species to species, and there are 13 categories of vitamins in humans. Among these, vitamins A, D, and E have been investigated regarding their influence on PCa prevention [67].

#### 2.4.1. Vitamin A

Vitamin A (also known as retinol or carotene) is found in liver, eggs, and vegetables. Essentially, vitamin A and its derivatives are required for sustaining vision and early development [68]. The relationship between PCa and vitamin A, particularly lycopene, a type of carotene, has been heavily studied due to its ability to prevent PCa. Three meta-analyses suggest that dietary intake of tomatoes or lycopene lowered PCa risk [69,70,71], and a recent a meta-analysis of 42 studies reported that not only dietary intake (RR = 0.88, 95% CI = 0.78–0.98) but also circulating concentrations (RR = 0.88, 95% CI = 0.79–0.98) of lycopene were significantly associated with reduced PCa risk according to dose-response relationships [69]. Lycopene in tomatoes is suggested to be the factor most likely associated with reduction of PCa risk reduction by vitamin A.

Although the anticancer activities of vitamin A are mainly associated with their antioxidant activities [72,73], other mechanisms have been proposed [74,75,76]. An animal study showed that dietary tomato and lycopene intake reduced the expression of genes involved in androgen metabolism/signaling pathways (*Srd5a1*, *Srd5a2*, *Pxn*, and *Srebf1*) in transgenic adenocarcinoma of the mouse prostate (TRAMP) mice [76]. However, there were variations in the activity of carotenoid cleavage enzymes, such as β-carotene 9′,10′-oxygenase 2 (BCO2), and the abilities of lycopene to inhibit PCa carcinogenesis were significantly attenuated in TRAMP *Bco2*^−/−^ mice relative to those in TRAMP *Bco2* wild-type mice [74]. These results suggest that genetic variations influence the anticancer activities of lycopene and its antioxidant activity and androgen signaling pathways.

#### 2.4.2. Vitamin D

Vitamin D is synthesized from a derivative of cholesterol by organisms exposed to sunlight; however, humans can easily experience vitamin D deficiency, and must obtain it from supplements or vitamin D-rich food, such as fish, dairy products, and mushrooms. The primary active form of vitamin D [calcitoriol (1,25 dihydroxyvitamin D3)] is a hormone that regulates the metabolism of calcium and phosphorus [68], with calcitriol shown to induce differentiation of immune cells and reduce cancer proliferation [77]. Additionally, calcitriol administration reduced PSA levels by >25% in 20% of patients with localized PCa after 3 months of treatment [78]. Despite the expected benefit of vitamin D for preventing PCa, the results of epidemiological studies are debatable, as some studies indicate that higher vitamin D levels negatively affect PCa status [79,80], whereas others suggest the exact opposite [81,82,83]. Furthermore, a meta-analysis of 25 studies provided little evidence to support a major role for vitamin D in preventing PCa development and progression [84].

In an animal study using TRAMP mice, calcitriol treatment slowed the progression of the primary tumor, as indicated by reduced prostate weight at 18 weeks of age; however, calcitriol significantly increased the number of distant organ metastases at 20 to 25 weeks of age [85]. Vitamin D likely exhibits both beneficial and adverse effects depending on the dose or duration of administration, with this likely reflected by controversial results in epidemiological studies.

#### 2.4.3. Vitamin E + Selenium

Vitamin E includes tocopherols and tocotrienols, and depending on their presence or absence and the position of the methyl group, they are further classified into four different isoforms: α, β, γ, and δ. Vegetable oils contain high amounts of vitamin E [86]. In particular, soybean, sunflower, corn, walnut, cottonseed, palm, and wheat-germ oils are rich in vitamin E, with the proportions of its isoforms varying depending on the type of oil.

Vitamin E exhibits strong antioxidant, anti-inflammatory, and anti-thrombolytic effects, and preclinical investigations suggest that it inhibits tumor growth [87]. A large prospective study of 29,000 men on the efficacy of vitamin E in reducing lung cancer risk concluded that vitamin E intake did not affect the risk of lung cancer but reduced PCa incidence [88]. However, since the release of those results, epidemiological studies have reported no association between vitamin E supplementation and PCa risk [89,90,91].

Further, a large intervention study [(Selenium and Vitamin E Cancer Prevention Trial (SELECT)] examined the effect of supplementation of vitamin E and selenium, finding that selenium + vitamin E supplementation had no effect on reducing PCa risk, although, surprisingly, vitamin E supplementation alone was associated with an increased risk of PCa (vitamin E group; HR = 1.17, 99% CI: 1.004–1.36) [92].

Recent studies focused on differences between the effects of different isoforms of vitamin E on PCa, revealing that δ-tocopherol inhibits PCa progression by attenuating AKT activation via phosphorylation at T308 in PCa cell lines and prostate-specific *Pten*^−/−^ mice [93,94]. Additionally, δ-tocotrienol reportedly inhibits PCa cell proliferation in vitro [95,96,97]. δ-Tocotrienol exhibits antitumor activity via endoplasmic reticulum stress, which induces vacuolation, and autophagy pathways. In many previous clinical studies, including SELECT, α-tocopherol was supplied as vitamin E, which might explain why vitamin E showed no effect on reducing PCa risk in those studies.

### 2.5. Polyphenols

Polyphenols are the most abundant and vital plant metabolites and are present in different proportions in most plants. Unlike vitamins, polyphenols are not essential for human survival but exhibit various biological activities, mainly antioxidant and anti-inflammatory. Polyphenols have been studied for their beneficial effects on human health, such as treatment of hypertension and prevention of cancer or cardiovascular disease [98]. The association of PCa with catechin and isoflavone is well documented.

#### 2.5.1. Catechin

Catechin is found mainly in green tea, with epigallocatechin-3-gallate (EGCG) the most commonly identified catechin in green tea (>50% of the total polyphenol content) and exhibiting a strong physiological activity [99]. Previous studies report inhibitory effects of EGCG on PCa progression and its molecular targets *in vivo* and *in vitro*.

EGCG shows effects against both androgen-sensitive and -insensitive human PCa cells, resulting in dose-dependent G0/G1-phase arrest and induction of apoptosis [100]. A study indicated that although arachidonic acid stimulated cell growth and increased prostaglandin E2 levels, EGCG attenuated these effects in both androgen-sensitive and -insensitive human PCa cells [101]. That study found that EGCG reduced levels of COX-2 at both the mRNA and protein levels in human PCa cells [101]. Another study reported that oral intake of EGCG suppressed PCa progression in 12-week-old TRAMP mice but not in 28-week-old mice, suggesting that EGCG suppressed early-stage PCa [102]. The authors also indicated that EGCG treatment downregulated IGF-1-related signaling (via IGF-1 and extracellular-regulated protein kinase) and COX-2 levels [102]. These findings suggest that EGCG acts through multiple mechanisms to induce cell cycle arrest and apoptosis of PCa cells and promotes a mechanism associated with COX-2-mediated anti-inflammatory effects.

Many epidemiologic studies have explored the association between tea intake and PCa [103,104,105,106,107], with inconsistent results regarding the benefits of tea. Notably, almost none of the studies categorized the tea (black vs. green) and included populations that consume black tea, which might not accurately reflect the association between green tea intake and PCa. A large-scale prospective study of 49,920 Japanese men including a high prevalence of green tea drinkers (Japan Public Health Center-based Prospective study: JPHC study) examined the relationship between consumption of green tea and PCa risk [108], revealing that men who drank five or more cups of green tea daily had a lower risk of advanced PCa relative to those drinking less than one cup daily (RR = 0.52, 95% CI = 0.28–0.96). Additionally, a meta-analysis of 10 epidemiological studies on green tea and PCa incidence reported that PCa risk decreased in a dose-dependent manner along with increased green tea consumption, with a significant risk reduction for those who consumed more than seven cups daily (RR = 0.81, 95% CI = 0.67–0.97 for 7 cups/day; RR = 0.74, 95% CI = 0.59–0.93 for 9 cups/day; RR = 0.56, 95% CI = 0.35–0.92 for 15 cups/day) [99]. These results indicate that green tea has an inhibitory effect on PCa carcinogenesis but must be consumed in large amounts to be effective.

#### 2.5.2. Isoflavones

Isoflavones are abundant in most leguminous plants. Additionally, soy isoflavones and their derivatives, genistein and daidzein, reportedly show efficacy in preventing PCa, with a study showing a reduced risk of PCa associated with soy isoflavone intake [109]. Soy isoflavones are similar in structure to 17β-estradiol; therefore, they can bind to the estrogen receptor (ER) and act as phytoestrogens. In particular, the binding affinity to ER-β is stronger than that to ER-α and comparable with that of 17β-estradiol. Additionally, soy isoflavones are more likely to induce the transcriptional activity of ER-β than of ER-α. The intensity of expression of ER-α or -β varies among different tissue types, with prostate and bone expressing higher levels of ER-β. Therefore, isoflavones are more likely to exhibit estrogenic effects in prostate tissue [109]. In addition to estrogenic effects, isoflavones reportedly exert antioxidant and inhibitory effects on tyrosine kinase activity [110,111]. An in vitro study reported that genistein induces apoptosis of PC3 cells, and that the mechanism involved suppression of nuclear factor-κB through the AKT signaling pathway [112].

In a study using mice as an orthotopic human PCa cell-transplant model, dietary genistein suppressed cancer metastases by 96% but did not alter tumor growth [113]. The authors showed that genistein increased the levels of proteins (e.g., protein tyrosine kinase 2 and p38 mitogen-activated protein kinase) promoting PCa progression, but decreased their phosphorylation [113]. Moreover, dietary genistein reduced the development of PCa in a dose-dependent manner in TRAMP mice [114]. Although there is general agreement that soy isoflavones, especially genistein, have an inhibitory effect on PCa progression, a low-dose genistein diet (250 mg/kg) promoted PCa growth and metastases as compared with a control and high-dose genistein diet (1000 mg/kg) in TRAMP-FVB mice [115]. In clinical trials, since isoflavones may also exert biphasic effects, similar to those in animal studies, their inadequate intake may lead to PCa progression. Therefore, caution should be exercised when setting the dose.

A randomized control study of 158 men in eight Japanese centers using a purified isoflavone supplement reported no significant change in the PSA value before and after treatment, and the incidence of PCa showed no significant difference between isoflavone and placebo groups, although for patients aged >65 years, PCa incidence in the isoflavone group was significantly lower (28.0% vs. 57.1%) [116]. Reports of isoflavone supplementation in patients with PCa include multiple randomized controlled trials that suggest that isoflavones show a suppressive effect on PCa progression based on biomarkers, such as serum PSA and testosterone levels [117,118,119,120], although the results have been inconsistent [121]. Such inconsistencies might be related to differences in dosage, duration, and dietary intake by the cohorts in each study.

## 3. Dietary Pattern

As noted, human studies comparing the consumption of individual nutrient factors or the presence or absence of vitamin or polyphenol supplementation often result in unclear outcomes. This might be due to significant differences in the original diet and lifestyle, among other factors, such as region, age, and race. Interactions and synergies between nutrient factors might also need to be considered to determine the effects of diets on PCa. Therefore, some studies report using cohorts that were classified and compared according to dietary pattern rather than individual nutrient factors. Typical dietary patterns include “Western” and “Prudent”. The “Western pattern” is characterized by a higher consumption of red meat, processed meat, potatoes, and high-fat dairy products, whereas the “Prudent pattern” is characterized by a higher intake of vegetables, fruits, legumes, and fish [122].

The JPHC study recently reported a comparison of PCa risk according to three dietary patterns: “Prudent”, “Western”, and “Traditional” patterns. The “Traditional dietary pattern” involves a characteristic Japanese diet that included high amounts of pickles, seafood, chicken, and Japanese sake [123]. This study found that the “Westernized dietary pattern” was associated with an increased risk of PCa in Japanese men (HR = 1.22, 95% CI = 1.00–1.49), and that the “Prudent dietary pattern” was associated with a lower incidence of PCa when restricted to cases detected by subjective symptoms (HR = 0.71, 95% CI = 0.50–1.02). With respect to the relationship between the “Western pattern” and PCa, although several epidemiological studies reported similar results in Argentina [124], Uruguay [125], Western Australia [126], and Iran [127], two cohort studies from western countries reported no association [128,129]. As for the “Prudent pattern,” a case-control study of 50 Iranian men with PCa reported that the “Healthy pattern” (i.e., “Prudent pattern”) reduced the risk of PCa, similar to the study in Japan [127]. However, studies in other countries reported no association between the “Prudent pattern” and PCa risk.

Epidemiological studies show no definitive conclusion regarding the relationship between dietary patterns and PCa risk, similar to many human studies focusing on single nutrient factors, because no studies worldwide have focused on multiple regions with widely different dietary patterns, and it is difficult to conduct a meta-analysis owing to inconsistent definitions of “dietary pattern.” In particular, the lack of an acceptable definition of a “dietary pattern” is a major issue, and previous studies have reported markedly different definitions. For example, the “Traditional dietary pattern” used in the study in Japan [123] was very different from the “Traditional pattern” used in the study in Argentina [124], where the dietary pattern more closely resembled that of the “Western pattern.” Furthermore, the lack of definition promotes arbitrary classification of patterns to suit a specific conclusion. Therefore, it is necessary to define a recognized classification of dietary pattern prior to conducting further research using the same classification.

## 4. Gut Microbiota

A large number of commensal microorganisms form colonies and a distinctive flora (i.e., microbiota) on all body surfaces of animals, including humans. In particular, the large intestine is an optimal environment for the abundant growth of diverse microorganisms (i.e., gut microbiota) [130]. The composition of gut microbiota is influenced not only by lifestyle (i.e., dietary pattern, smoking, and exercise) but also by maternal transfer, age, and antibiotic use. Dietary composition, an element of lifestyle, has a direct and profound effect on gut bacteria [131]. The Western diet (high fat/high sugar) reduces the diversity of gut microbiota in mice, resulting in an increase in *Bacteroides* species and *Ruminococcus torques* [132]. A study comparing the fecal samples of children in Burkina Faso (eating a high-fiber diet) with European children (eating a typical Western diet) found that the gut microbiota of children from Burkina Faso contained significantly less *Enterobacteriaceae* (*P* < 0.05), and that the stool contained significantly more short-chain fatty acids (*P* < 0.001), one of the metabolites generated by bacteria [133].

The interaction of microbiota with the host has profound effects on immunity and metabolism, and these interactions contribute to the maintenance of host–microbe homeostasis [134,135]. In recent years, the comprehensive analysis of the microbial genomes and metabolites of microbiota has become easier and more accurate due to the enhanced performance of equipment, such as next-generation sequencers and mass spectrometers [136]. Additionally, there is increasing interest regarding host interaction with gut microbiota based on the increased accessibility introduced by advances in technology. Interestingly, studies suggest that gut microbiota is involved not only in intestinal diseases but also in diseases in other organs, with several laboratory and human studies suggesting its involvement in Alzheimer’s disease [137,138], rheumatoid arthritis [139,140], and diabetes [141,142]. Additionally, the influence of gut microbiota on cancer is a research area that has received substantial attention based on findings that gut microbiota, which is altered by various external factors, including dietary composition, is involved in all stages of cancer, including initiation, progression, treatment outcomes, and adverse reactions [143].

Numerous studies report an association between gut microbiota and intestinal cancer, showing direct effects by bacteria in the gut; however, few have reported an association between gut microbiota and cancer in other organs, particularly those that are not directly related to the gut (e.g., the prostate). In colorectal cancer, *Fusobacterium nucleatum* promotes carcinogenesis by generation of a proinflammatory microenvironment, as shown by multiple basic research and human studies performed on various continents [144,145]. Moreover, multiple studies have addressed the effects of bacterial components and metabolites on cancer, as well as the influence of lipoteichoic acid and lipopolysaccharides (components of the outer membrane of Gram-positive and -negative bacteria) or butyrate (a type of short-chain fatty acid produced by enteric bacteria that ferment dietary fiber) [146,147,148,149].

A previous study of the gut microbiota of 133 patients undergoing prostate biopsies in the United States showed an association between the presence or absence of PCa and microbial composition, and that *Bacteroides* and *Streptococcus* spp. were significantly enriched in the gut microbiota of patients with PCa [150]. This suggests that gut microbiota might be involved in not only gastrointestinal cancers but also PCa. Because the composition of gut microbiota differs greatly according to region [151], it remains unclear how gut microbiota (or a particular bacterial species) is involved in PCa for a given population. Furthermore, the mechanisms by which gut microbiota control PCa have not been elucidated. Although the prostate is not an organ directly affected by gut microbiota, the prostate could be affected indirectly by cytokines and immune cells modified by microbiota in the gut or bacterial metabolites and components absorbed from the intestine that enter systemic circulation (i.e., a “microbiota-gut-prostate axis”).

These findings suggest the potential influence of diet and nutrition on PCa and its partial mediation by gut microbiota. We anticipate that future studies will reveal more about the “microbiota-gut-prostate axis”.

## 5. Conclusions

The effects of diet and nutrients on PCa pathogenesis and progression have received increasing attention. Animal studies have reported that certain nutrients, including fat, protein, carbohydrates, vitamins (vitamin A, D and E), and polyphenols, are indeed involved in PCa pathogenesis, and progression through several mechanisms, including inflammation, antioxidant effects, and the effects of sex hormones. However, contrasting findings of clinical studies have deterred determining which nutrients have a positive or deleterious effect on PCa incidence and/or progression. Weak effects of single nutrients and interactions among different nutrients have resulted in controversial results among clinical studies conducted among residents with different dietary backgrounds. Therefore, it is important to assess the effect of dietary patterns that comprehensively summarize nutrient intake. It is generally believed that a healthy dietary pattern (e.g., low in meat and high in vegetables) can help prevent PCa and lifestyle-related diseases; however, no evidence is available regrading this belief. Future studies are expected to establish accepted definitions of dietary patterns and conduct a large-scale cohort study targeting multiple regions (Table 1).

Recent studies suggest that the diversity of gut microbiota affects various diseases, and the attention given to research involving gut-microbiota composition has increased. Therefore, the diversity of gut microbiota might account for differences in the influence of diet and nutrition on PCa. These interactions between gut microbiota and nutrients, where nutrients alter the composition of gut microbiota, thereby altering metabolic pathways and/or nutrient absorption, might generate diverse responses to PCa. We believe that gut microbiota is the key to further understanding the influence of diet and nutrition on PCa, and an intervention of dietary patterns could contribute to the prevention of PCa through the change in microbiota.

## Figures and Tables

**Table 1:** Summary of nutrients, dietary patterns, and evidence for their association with prostate cancer.

Subject	Type	Source Examples	Risk	Reported Mechanisms	References
Fat	Total fat	Animal meat, butter	Increased PCa risk	IL-6/STAT3 pathway MDSC, Macrophage infiltration GF-I signaling pathway Increase in local androgen	[17,18,19,20,21,22,23,24,25,26,27,28,29,30,31,32,33,34,35,36,37,38,39,40,41,42,43]
	Unsaturated fatty acid	Fish, vegetable oil	Decreased PCa risk (probable)	Decrease in estradiol, testosterone, and androgen receptors	
Protein	Total protein	Animal meat, soy, dairy product,	No change in PCa risk	N/A	[45,46,47,48,49,50,51,52,53,54,55,56,57,58]
	Heterocyclic amine	Well-cooked meat	Increased PCa risk	Mast cell, Macrophage infiltration	
	Dairy product	Milk, cheese	Increased PCa risk (probable)	N/A	
Carbohydrate		Fruit, rice, potato, sugar	Increased PCa risk (probable)	IGF-I signaling pathway	[28,60,61,62,63,64,65,66]
Vitamin	VitaminA (Lycopene)	Tomato	Decreased PCa risk	Decrease in androgen metabolism Antioxidative effect	[69,70,71,72,73,74,75,76]
	VitaminD (Calcitoriol)	Fish, dairy product, mushroom	Decreased PCa risk (controversial)	Promotion of immune cell differentiation	[78,79,80,81,82,83,84,85]
	VitaminE (Tocopherol)	Vegetable oil	No change in PCa risk (controversial)	Anti-inflammatory effects Endoplasmic reticulum stress Antioxidative effect	[88,89,90,91,92,93,94,95,96,97]
Polyphenol	Catechin	Green tea	Decreased PCa risk	IGF-I signaling pathway COX-2-mediated anti-inflammatory effects	[99,100,101,102,103,104,105,106,107,108]
	Isoflavone	Legume	Decreased PCa risk (probable)	Estrogenic effects Antioxidative effect Inhibition of tyrosine kinase Suppression of NFκB	[109,110,111,112,113,114,115,116,117,118,119,120,121]
Dietary pattern	Western pattern	Meat, potato, dairy product	Increased PCa risk (controversial)	N/A	[123,124,125,126,127,128,129]
	Prudent pattern	Vegetable, fruit, legume, fish	Decreased PCa risk (controversial)	N/A	
